# Complete mitochondrial genome of Grey Treepie, *Dendrocitta formosae* (Aves: Corvidae)

**DOI:** 10.1080/23802359.2019.1629353

**Published:** 2019-07-16

**Authors:** Da-Wei Liu, Sun Cheng-He, Fei Yi-Ling, Sen-Lin Hou, Tang Song-Ze

**Affiliations:** a Nanjing Forest Police College, Nanjing, China;; b Key Laboratory of Wildlife Evidence Technology State Forest and Grassland Administration, Nanjing, China;; c College of Biology and the Environment, Nanjing Forestry University, Nanjing, China

**Keywords:** Grey Treepie, mitochondrial genome, *Dendrocitta formosae*

## Abstract

We report the complete mitochondrial genome of *Dendrocitta formosae*. The genome is a closed circular molecule of 16,875 bp, with all genes exhibiting typical avian gene arrangement. The overall base composition of this species’ mitogenome is 24.33% T, 30.49% C, 30.17% A, and 15.01% G. The A + T content is 54.50%. Phylogenetic analysis of the complete mitogenome of 12 species conducted using the neighbour-joining method and kimura 2-parameter model suggested that the mitogenome of *D. formosae* was the closest to that of *Pyrrhocorax graculus* and *P. pyrrhocorax.* The results could aid future studies on *Dendrocitta* and *Pyrrhocorax* molecular evolution and phylogeny.

*Dendrocitta formosae*, belonging to the family Corvidae (Aves, Passeriformes), is distributed in Bangladesh, Bhutan, China, India, Myanmar, Nepal, Pakistan, Thailand, Vietnam, and Laos (IUCN 2019). This species inhabits forests, shrublands, and arable lands (Mackinnon and Phillipps 2000). Because of the on-going habitat destruction on Hainan, China, the current population of this bird is declining (IUCN 2019). Relatively scarce information is available on the complete mitogenome of *Dendrocitta* species. Herein, we describe the complete mitogenome of *Dendrocitta formosae*.

We characterized the complete mitogenome of a *D. formosae* individual sampled in Jiangning District, Nanjing City, China (31.89°N, 118.76°E). Whole blood sample for genomic DNA extraction was collected from the individual and stored in the Forensic Identification Center for Forest Police of the State Forest Bureau. Genomic DNA was extracted using the DNAiso reagent (Takara, Beijing, China). A set of primers were designed for PCR, and Sanger sequencing based on the complete mitogenomes of *Urocissa caerulea* (GenBank accession: MG932654.1), *Urocissa erythrorhyncha* (GenBank accession: JQ423932.1), *Pica pica* (GenBank accession: HQ915867.1), and *Corvus frugilegus* (GenBank accession: Y18522.2).

The complete mitochondrial genome (GenBank accession: MK875763) of *D. formosae* is a typical circular DNA molecule of length 16,875 bp. The nucleotide composition is 24.33% T, 30.49% C, 30.17% A, and 15.01% G, with A + T content of 54.50%. The mitogenome consists of 13 protein-coding genes, 22 transfer RNA genes, two ribosomal RNA genes, and one control region. The structure of the mitogenome is identical to that of most avian species (Caparroz et al. 2018; Liu et al. 2019; Sun et al. 2019).

Phylogenetic analysis of *D. formosae* was performed based on the complete mitogenome of 11 other birds. Sequence dataset was aligned using ClustalX and analyzed using the neighbour-joining method and the kimura 2-parameter model in MEGA 7.0, with 1000 bootstrap replicates (Kumar et al. 2016). The phylogenetic tree showed that the mitogenome of *D. formosae* was genetically the closest to that of *Pyrrhocorax graculus* and *P. pyrrhocorax* (Figure 1), which is in accordance with the current morphological classification. The genome information obtained herein could contribute to future studies on the molecular evolution and phylogeny of *Dendrocitta* and *Pyrrhocorax.*

**Figure 1.**
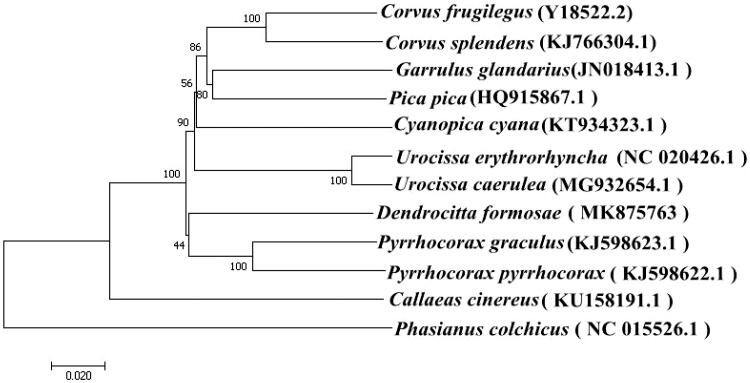
Neighbour-joining phylogenetic tree based on the complete mitogenomes of 12 avian species, constructed using MEGA 7.0.
